# Structural Characterization of Receptor–Receptor Interactions in the Allosteric Modulation of G Protein-Coupled Receptor (GPCR) Dimers

**DOI:** 10.3390/ijms22063241

**Published:** 2021-03-22

**Authors:** Raudah Lazim, Donghyuk Suh, Jai Woo Lee, Thi Ngoc Lan Vu, Sanghee Yoon, Sun Choi

**Affiliations:** Global AI Drug Discovery Center, College of Pharmacy and Graduate School of Pharmaceutical Sciences, Ewha Womans University, Seoul 03760, Korea; raudah@ewha.ac.kr (R.L.); dsuh@ewha.ac.kr (D.S.); jwl123@ewha.ac.kr (J.W.L.); vutnl@ewhain.net (T.N.L.V.); caddshyoon@ewhain.net (S.Y.)

**Keywords:** G protein-coupled receptor (GPCR), dimerization, allosteric modulation, protein dynamics, receptor–receptor interaction, PPI prediction, protein dynamics, peptide design

## Abstract

G protein-coupled receptor (GPCR) oligomerization, while contentious, continues to attract the attention of researchers. Numerous experimental investigations have validated the presence of GPCR dimers, and the relevance of dimerization in the effectuation of physiological functions intensifies the attractiveness of this concept as a potential therapeutic target. GPCRs, as a single entity, have been the main source of scrutiny for drug design objectives for multiple diseases such as cancer, inflammation, cardiac, and respiratory diseases. The existence of dimers broadens the research scope of GPCR functions, revealing new signaling pathways that can be targeted for disease pathogenesis that have not previously been reported when GPCRs were only viewed in their monomeric form. This review will highlight several aspects of GPCR dimerization, which include a summary of the structural elucidation of the allosteric modulation of class C GPCR activation offered through recent solutions to the three-dimensional, full-length structures of metabotropic glutamate receptor and γ-aminobutyric acid B receptor as well as the role of dimerization in the modification of GPCR function and allostery. With the growing influence of computational methods in the study of GPCRs, we will also be reviewing recent computational tools that have been utilized to map protein–protein interactions (PPI).

## 1. Introduction

G protein-coupled receptors (GPCRs) belong to a large family of seven-transmembrane (TM) proteins with structural topologies defined by the general presence of the extracellular (EC) domain, the intracellular (IC) domain, and a TM domain comprising of seven helices that connects the EC and IC domains of the receptors. The TM domain serves as a conduit for the flow of information initiated by the binding of endogenous orthosteric ligands from the cell’s exterior and triggering the binding of cytosolic proteins such as the heterotrimeric guanine nucleotide-binding protein (G protein), GPCR kinases (GRKs), and β-arrestin within the cell. This process, being allosterically driven, spurred studies that aimed to understand the process of allosteric modulation in driving GPCR activation [1,2,3,4,5,6,7]. 

Structural studies have revealed the significance of conformational plasticity in the allosteric regulation of GPCR activity. The structural flexibility of GPCRs empowers the receptor family to cascade a variety of extracellular signals—spanning from photons to neurotransmitters and hormones—across the membrane, hence equipping GPCRs with the capacity to affect multiple signaling pathways. Depending on the G protein subtypes (G_s_, G_i/o_, G_q_, and G_12/13_) binding at the intracellular binding site, specific physiological functions ranging from taste, vision, and synaptic transmission are set in motion [8,9]. This versatile nature of GPCRs rendered them attractive as drug targets and opened numerous possibilities in the development of novel therapeutics for the treatment of a wide range of diseases and conditions [1,2,3,4,5]. While numerous experimental and computational studies have been conducted to examine the structural architecture and dynamics of GPCRs as monomers, these studies lead to a riveting question regarding the possibility of synergistic interactions between GPCRs to prompt specific signaling pathways. The growing number of studies investigating the role of dimerization and oligomerization in steering GPCR functions demonstrated the increasing interest in this topic despite its controversial status in the GPCR community [10,11,12,13].

Studies are emerging in support of GPCR homo/heterodimers and higher order oligomers, indicating the possibility of GPCRs to operate beyond the more congenial postulation of functional monomers [10,12,14,15,16,17,18,19,20,21,22]. The earliest allusive indication of GPCR oligomerization arose from kinetic binding assays performed by Limbird et al. for β-adrenergic receptors (β-ARs) on frog erythrocyte membranes [21]. In this study, the negative cooperativity between β-AR monomers on the membrane was inferred based on the different dissociation rates of ^3^H (-)alprenolol observed in two different conditions set apart by the surplus of unlabeled (-)alprenolol in one. Henceforth, the collection of indirect data from various traditional pharmacological and biochemical experiments such as binding assay, gel electrophoresis, immunoaffinity chromatography, chemical cross-linking, and co-immunoprecipitation studies further substantiated this phenomenon [22]. Recent explicit evidence reported the observation of various classes of GPCRs existing as homodimers, heterodimers, and/or higher-order oligomers through a variety of biophysical studies—single-molecule fluorescence-based approaches, X-ray crystallography, nuclear magnetic resonance (NMR) spectroscopy, and cryogenic electron microscopy (cryo-EM)—as well as computational studies. These have garnered more interests for the study of GPCR oligomerization, particularly for the potential implications to drug design and discovery [12,14,15,16,17,18].

**Figure 1:**
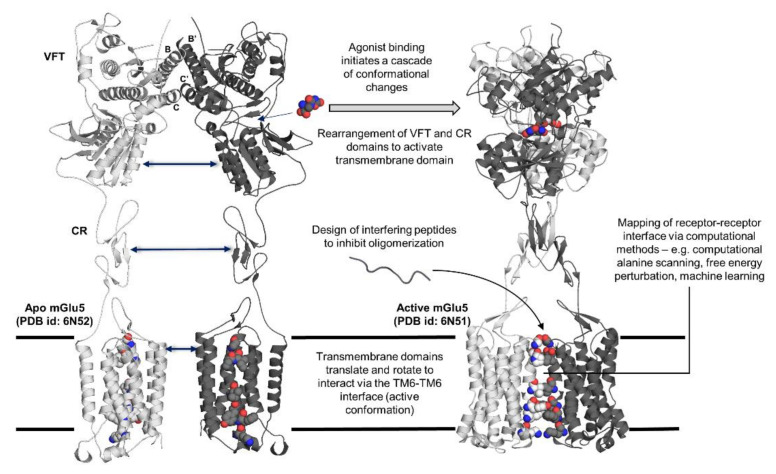
Summary of topics covered regarding receptor–receptor interactions in G protein-coupled receptor (GPCR) oligomers. Structural changes afforded through the binding of an agonist (L-quisqualate) to mGlu5 portrayed through X-ray crystal structure of mGlu5 in apo (PDB id: 6N52) and active (PDB id: 6N51) states. Helices B and C, which are involved in the stabilization of the dimer, are labelled. (VFT: Venus flytrap domain; CR: cysteine-rich domain).

In this review, we will focus on the structural aspect of the allosteric modulation of GPCR dimers, specifically for two well-characterized receptors, namely metabotropic glutamate receptor (mGluR) and γ-aminobutyric acid B receptor (GABA_B_R), both of which have their full-length structures recently solved. This review will also highlight studies that proposed the alteration of GPCR activity and allosteric modulation mechanism through dimerization—an interesting phenomenon that can be exploited to further boost the potential of GPCRs as a therapeutic target for new disease indications [23]. As available three-dimensional structures of GPCR dimers are limited in comparison to the number of dimers validated through experiments, the biophysical characterization of receptor–receptor interactions via computational methods have gained ground as a potential tool for the mapping of intra- and inter-subunit interactions at the receptor–receptor interface. Therefore, we will also highlight some current computational methods that have been or could be applied to investigate the protein–protein interface. Figure 1 illustrates an overview of the topics discussed in this review.

## 2. Role of Receptor–Receptor Interactions in the Allosteric Modulation of GPCR Activation

The comprehensive scrutinization of class A receptors has continuously supplied us with information on the structures and dynamics of the proteins, albeit the disproportionate distribution between inactive and active states solved. Nevertheless, advances in protein engineering and biophysical characterization techniques have propelled accessibility to the less solved active state configuration, allowing studies examining the structural disparity between the two states. The juxtaposition of the active and inactive configurations revealed compelling differences in highly conserved motifs known as the molecular switches that are conveyed to be important for allosteric communication between the distal ends of the TM domain, namely the orthosteric and intracellular protein binding sites. This forms the main cognizance of TM domain activation in the GPCR family. However, recent studies have established the presence of GPCR dimers across different classes of GPCRs. This discovery opens the possibility of TM domain activation being governed not just by long-range allosteric communication between the orthosteric and intracellular binding sites within a single receptor (cis-activation) but also through previously unprecedented pathways involving receptor–receptor interactions (trans-activation) [24]. This section will discuss the structural aspect of the mechanism governing the allosteric modulation of the trans-activation of two widely accepted GPCR dimers, namely mGluR homodimer and GABA_B_R heterodimer. This section will also highlight studies supporting the occurrence of dimerization involving class A and class C GPCRs, and it will briefly discuss how dimerization may alter the native activity of the receptor.

### 2.1. Class C GPCRs: A Potential Model for GPCR Trans-Activation

The concept of dimerization has been widely accepted for class C GPCRs, and cooperativity between protomers of this family of receptors—both positive and negative—has been proposed to be vital for signal transduction [25,26,27,28]. Several studies have been conducted to understand the mechanism governing the activation of class C GPCRs, specifically mGluR and GABA_B_R dimers. These studies inevitably led to insights pertaining to the allosteric regulation of signal transduction in GPCR dimers. Class C GPCRs have been proposed to be a potential model for the comprehension of allosteric regulation and cooperativity for other classes of GPCRs, albeit a tendentious comparison, since their sequences and overall structures differ from other classes. Nonetheless, several structural similarities with class A GPCRs have been drawn that uphold this comparison. 

The most significant similarity lies in the TM domains of class A and class C GPCRs. The similar topologies of the seven TM helices lead to a shared “ionic lock” feature that occurs between the intracellular regions of TM3 and TM6—a conserved “molecular switch” that when formed maintains the inactive conformation of class A GPCRs [26,29,30]. While a salt bridge between a conserved Arg^3.50^ and Glu(Asp)^6.30^ defines the ionic lock present in class A, this feature occurs via Lys^3.50^ and Glu^6.35^ in class C [31,32]. The numbers in superscript represent the Ballesteros–Weinstein numbering system in which the first digit indicates the TM helices 1 to 7 and the digits following the decimal (a separator) denote the residue position relative to a highly conserved residue within a single TM helix, which is assigned as residue 50 [33]. Site-directed mutagenesis performed at the aforementioned residues and a neighboring Ser613, in IC loop 1 (interacts with Lys^3.50^), to either stabilize or destabilize the ionic lock in class C GPCRs afforded a decrease or increase in the constitutive activation of their TM domains compared to wild type, respectively. This corroborated the analogous behavior of this motif in both GPCR classes [32]. Residues Lys^3.50^, Glu^6.35^, and Ser613 are also highly conserved in mGluR, GABA_B_R, calcium-sensing receptor, and T1R taste receptor, and mutations of these residues or others near the ionic lock reportedly altered the signaling pathways of class C GPCRs [31,32,34]. For instance, the point mutation of Glu^6.35^ to Lys in mGlu6 was reported to be the cause of congenital night blindness. This phenotype was expressed due to altered G protein signaling, causing the receptor to prefer G_i_ coupling over native G_o_ coupling [32,35]. The comparable TM topologies of the two classes of GPCRs was further evinced through homology models of class C GPCRs generated using the crystal structure of bovine rhodopsin [30,36,37]. These studies conducted afforded reliable observations that provided insights on the allosteric modulations of class C receptors [30,36,37,38]. 

MGluRs have also exhibited similar activation activity as rhodopsin-like receptors. Goudet et al. demonstrated this characteristic by examining the activity of the TM domain of a truncated mGlu5 (no Venus flytrap (VFT) and cysteine-rich (CR) domains) in the presence of a negative allosteric modulator (NAM) (MPEP; 2-methyl-6-(phenylethynyl)-pyridine hydrochloride) and a positive allosteric modulator (PAM) (DFB; 3,3′-difluorobenzaldazine) [39]. The binding of MPEP to the TM domain of the truncated mGlu5 led to the inhibition of the constitutive activity of the receptor relative to wild type. On the other hand, DFB binding resulted in the direct activation of the TM domain. While DFB has been classified as selective PAM with no agonistic effect on wild-type mGlu5, the absence of the VFT and CR domains permitted the ligand to behave as a full agonist, thus enabling receptor activation through a signaling pathway akin to that of a rhodopsin-like receptor [39,40]. A comprehensive analysis of the binding site of MPEP through site-directed mutagenesis and homology modeling of mGlu5 also discerned a binding pocket at the TM domain that coincides with the orthosteric binding site of rhodopsin [39]. Analysis of the three-dimensional structures of class C GPCRs solved in the presence of allosteric ligands further highlight this similarity [18,25,41]. These studies assert the similarities in the structural build of the TM domains of class A and C GPCRs, validating the potential of class C GPCRs to be a model system for the mechanistic study of TM domain activation of GPCR dimers in general.

### 2.2. Elucidation of Allosteric Modulation via Full-Length Structures of Class C GPCR Dimers

**Figure 2:**
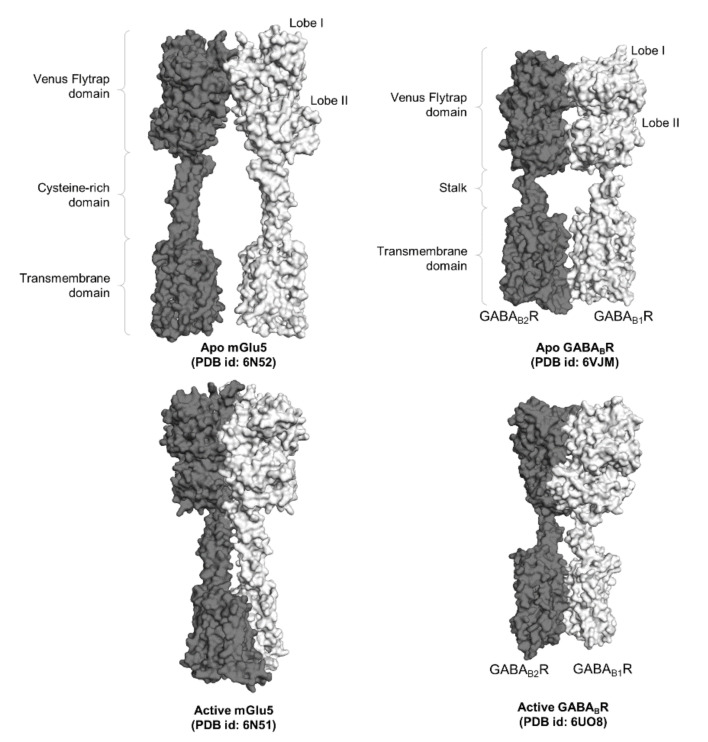
Surface representation of two full-length class C GPCR dimers, namely mGlu5 and GABA_B_R. The functional domains of the GPCR dimers, namely the Venus flytrap domains, the cysteine-rich domain in mGluR, the stalk in GABA_B_R, and the transmembrane domains, are labeled accordingly.

A structural feature that distinguished class C GPCRs from other classes is a large N-terminal EC domain that comprised of approximately 400 to 600 amino acids [26,28]. This domain encompasses a bilobed ligand-binding region that resembles a Venus flytrap; hence, it is also known as the VFT domain. The VFT domain comprises of two lobes, lobe I (N-terminal lobe) and lobe II, with a cleft in between that accommodates an agonist or an antagonist [25,28,42]. This large domain, with the exception of GABA_B_R, is connected to the TM domain via a CR domain [17,26,27,28]. Associations between lobes I of the VFT domains of partnering receptors in both inactive and active states engendered most class C GPCRs as obligate dimers, and this was structurally corroborated through the recently reported full-length apo structures of mGluR homodimer and GABA_B_R heterodimers in the “Roo” (Rest open–open) conformation [28,43,44,45,46] (Figure 2). The type of interactions established at this interface varies across class C GPCRs. Hydrophobic interactions and a nonessential, conserved disulfide bridge that formed between two flexible loops of the protomers are observed in mGluR homodimers, while GABA_B_R heterodimers are mainly stabilized through polar interactions [18,26,47]. 

A recent study by Koehl et al. combined data from X-ray crystallography, cryo-EM, and biochemical assays to examine the activation pathway of mGlu5 [28]. This study provided the first complete, three-dimensional structures of mGluR in both active and inactive states, thus allowing the scrutinization of the conformational plasticity of mGluR during activation. Ligand binding at both the VFT domains of the mGluR homodimer prompted a configurational change dictated by a less compact packing of two helices—named helices B and C (Figure 1)—in comparison to the apo structure. These helices bordered the interface between adjacent protomers at lobe I, and hydrophobic interactions are mainly established between conserved residues of these helices [26,28]. The more relaxed lobe I–lobe I interface promoted the formation of polar interactions near the apices of helices B leading to the stabilization of the “Acc” (Active close–close) conformation [28]. The concurrent activation of both VFT domains of mGluR homodimers is noted to be essential for optimal receptor activity, although the binding of an agonist at one of the VFT domains has been shown to partially activate mGlu5 receptor via an “Aco” (Active close–open) conformation [18,47].

The comparison of the active and inactive states of mGlu5 revealed that the TM domains moved closer together and undergo a 20° rotation to adopt an active conformation characterized by a TM6–TM6 interface [28,43]. This maneuver, mediated by interactions established between the CR domain and EC loop 2 (ECL2) of the TM domain, was speculated to be vital, as it aids in the translation and rotation of the TM domains that enabled the formation of specific inter-subunit interactions that could ameliorate the activity of mGluR [28,29,48,49]. Observations revealed through the three-dimensional structure of mGlu5 were also congruent with earlier experimental studies, all of which emphasized the importance of both intra- and inter-subunit in modulating allosteric communication between the VFT and the TM domains [25,29,49,50,51].

In addition to the mGlu5 homodimer, several structures of the full-length metabotropic GABA_B_R heterodimer have also been solved [43,44,45,46]. Shaye et al. reported the structures of four full-length GABA_B_R in the active and inactive states as well as two intermediate states. In this study, they have combined the use of cryo-EM as well as molecular dynamics (MD) simulations to elucidate the intricate dynamics of GABA_B_R activation [45]. GABA_B_R forms an obligate heterodimer comprising of two different subunits, namely the GABA_B1_R (GB1) and the GABA_B2_R (GB2). In addition to association at the VFT domains, stabilization of the heterodimeric apo form was also assisted through polar interactions established at the intracellular segments of TM3 and TM5 of GB2 and GB1, respectively. Similar to mGluR, allosteric modulation originates from the orthosteric binding site of the VFT domain and engenders a cascade of conformational changes leading to the activation of the TM domain. In other respects, the GABA_B_R heterodimer follows a distinctive signal transduction mechanism in which the agonist only binds to the VFT domain of GB1, and G protein activation proceeds via the activation of the TM domain of GB2 [45,52,53,54,55,56]. 

The contrasting ligand-binding competence of the subunits rendered GABA_B_R an attractive model for the study of asymmetric trans-activation [53,57]. With the availability of the three-dimensional structures of the intermediate states of GABA_B_R, the allosteric pathway leading to the initiation of downstream signaling via GB2 could be harnessed. The two intermediate states solved for GABA_B_R also evinced the dynamic nature of receptor activation. Ligand-binding at GB1 was proposed to have created an equilibrium between the partially (Int-1) and fully closed (Int-2) conformations of its VFT domain [45]. The partially closed conformation of the VFT domain of GB1 induced the rotation of both GB1 and GB2, which brings the two protomers closer together, while keeping lobes II of GB1 and GB2 far apart. In this state, the TM domains were oriented in the inactive TM5–TM5 topology, albeit no interaction was established between the two helices. As GB1 transitions to the fully closed configuration at the VFT domain, lobes II of the GB1 and the GB2 subunits gravitated toward each other. This conformational change induced signals that descend a connecting “stalk” (Figure 2), leading to the characteristic active TM6–TM6 topology necessary for class C GPCR activation [26,28,43,45,58]. With available crystal structures, further computational studies of GABA_B_R could furnish us with insights related to the dynamics of negative cooperativity in driving asymmetric G protein signaling, which is a characteristic that has been commonly reported in GPCR dimers [59,60,61]. 

### 2.3. Altered GPCR Activities Induced through Heterodimerization

Even though the homodimerization of mGluR has been widely acknowledged to regulate neuronal function, the existence of mGluR heterodimers is still as debatable as the concept of dimerization for other GPCR families. Even so, the presence of several mGluR heterodimers has been alluded through experimental studies [51,62]. The formation of the heterodimeric complex between Group I mGluRs, namely mGlu1 and mGlu5, at the hippocampal neurons has been verified by Pandya et al. through a series of immunoprecipitation experiments [62]. The tendency of this dimer to exist as a functional heterodimer and contribute to signal transduction was subsequently verified by Werthmann et al. through functional complementation experiments in HEK293 cells [51]. Additionally, the mGlu1/5 dimer was also proposed to afford a distinct allosteric modulation pathway in comparison to their homodimeric counterparts. The MGlu1/5 dimer follows the symmetric signaling (equal probability for both protomers to engage G protein) exhibited by their respective homodimers. However, the receptor’s response to G protein coupling is dependent on the protomer that the intracellular protein engages, and the activation of both protomers is necessary for G protein activation. This observation contradicts the activation pathway observed in their respective homodimers, whereby the inhibition of one protomer did not curtail G protein activation [63]. 

The MGlu2/4 dimer has also been identified in vivo and is one of the most studied mGluR heterodimers [59,64]. Unlike their respective homodimers and mGlu1/5 dimer, the mGlu2/4 heterodimer follows an asymmetric activation pathway, which entails selective G protein binding to mGlu4 [59]. However, when mGlu4 is stabilized in its inactive state via NAM binding or when a PAM is bound to the TM domain of mGlu2, the mGlu2/4 dimer adopted an alternative activation profile via G protein coupling at mGlu2. Asymmetric cooperativity has also been found to be ubiquitous for heterodimeric pairs comprising of mGlu2 and other Group II (mGlu3) and Group III (mGlu4, mGlu6-8) mGluRs [59,60,61]. The binding of G protein to only one protomer is also a mechanism that has been evidently adopted by most GPCR homodimers and heterodimers despite differences in the allosteric modulation pathway. This observation iterates the importance of negative cooperativity between the TM domains of GPCR dimers through which the activation of one protomer blocks the signaling capability of the other, directing G protein coupling to a single protomer. Positive cooperativity between TM domains was also observed in mGlu2/4 and mGlu1/5 dimers. In this case, the inactive state of one protomer initiated the activation of the other through positive allosteric effects [51,63,65]. 

While the structures of class A and C GPCRs differ considerably as a whole, the TM domains of these receptors share similar topologies (vide supra) leading to the possibility of class A GPCRs existing as dimers. The acquiescence of class A GPCR dimerization is also stimulated through experimental evidence of their physical interactions with mGluRs and other class A receptors. Numerous studies conducted to understand the physiological aspect of class A/class C GPCR heterodimers have associated heterodimerization to the modification of the receptor’s function, trafficking, and pharmacology [12,19,26,66]. While the mechanism controlling the dimerization process is still unclear and research have afforded diverse explanations for their assemblies, physical interactions between class A and class C GPRCs have been reported, evincing the formation of heterodimers. These heterodimers include mGluR/serotonin 5-HT_2A_ receptor (5-HT_2A_R), mGlu5/adenosine A_2A_ R (A2AR), Glu5/dopamine D1 receptor (D1R), and mGlu5/mu-opioid receptor (MOR) [26,67,68,69,70,71,72]. Among these heterodimers, mGlu2/5-HT_2A_R is the most widely investigated and association to the pathophysiology of psychosis in schizophrenia and Parkinson’s disease, as well as dyskinesia in the latter rendered this heterodimer an attractive target for the treatment of these diseases [68,69,70,73]. 

## 3. Computational Methods Utilized for the Understanding of Receptor–Receptor Interactions in GPCR Dimers

Over the recent years, the three-dimensional structures of GPCRs have become more accessible in conjunction with the continuous improvements in structural biology [11,25,74]. Through the application of modern computational tools, the conformational plasticity of GPCRs can be investigated in an environment that replicates their native surroundings, hence providing a more realistic representation of the receptor [75,76,77]. Access to the structures of GPCR dimers, especially class C GPCR obligate dimers, has empowered researchers to explicate the conformational transitions critical for the allosteric modulation of GPCR trans-activation (vide supra) and to resolve protein–protein interactions (PPIs) that aid in their stabilization in the active and inactive states. However, the structural and mechanistic information available for GPCR dimers are still limited due to the smaller number of solved GPCR dimers in the Protein Data Bank as compared to the aggregate of dimers uncovered through experimental studies. This shortcoming has led to the development of modern computational tools that permitted the mapping of receptor–receptor interactions through computer algorithms and the use of protein models to predict hotspots and inter-residue interactions at the protein–protein interface. These tools could provide information to experimentalists for the design of GPCR variants that are stabilized in their dimeric form, allowing their crystallization. In this section, we will highlight some current computational methods that have been or may be applied to investigate the receptor–receptor interface of GPCR dimers.

### 3.1. Hot-Spot and Interface Interaction Discovery Using Computational Methods

**Figure 3:**
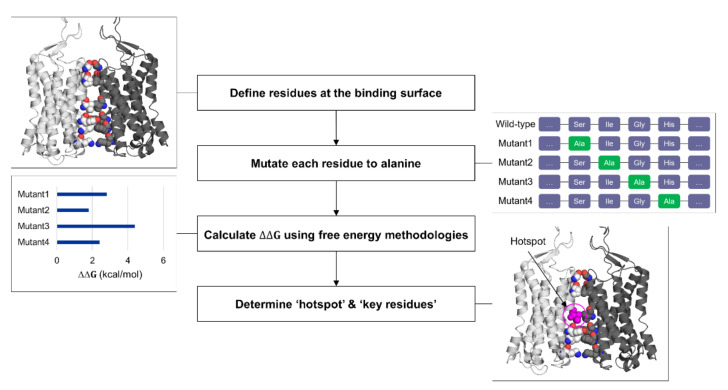
General workflow for computational alanine scanning (CAS).

To identify the “hotspots” at the protein–protein interface and measure the binding affinity, alanine scanning mutagenesis combined with binding free energy calculations can be utilized [78]. Since verifying key residues at the interface can be immediately fruitful with fast-developing protein engineering technologies, elucidating “hotspots” at the binding site has become more attractive [79]. Alanine scanning mutagenesis, a site-directed mutagenesis into chemically inert alanine, has been extensively used to gauge the importance of specific residues upon protein function and stability [80]. Then, the method started to be used computationally (CAS, computational alanine scanning) to reduce time and cost [81]. To compensate for the loss in the accuracy compared to the experimental counterpart, free energy methodologies have been applied to CAS (Figure 3). A number of free energy methods to determine the binding affinity of two bodies are available with the trade-off between computational efficiency and accuracy [82]. The rigorous yet expensive methods are long-timescale MD simulations with kinetic/thermodynamic analysis using machine learning applications, and free energy perturbation and thermodynamic integration with biasing potentials [83,84]. Relatively cheaper ones include molecular mechanics combined with Poisson–Boltzmann or generalized Born surface area solvation (MM/PBSA or MM/GBSA), Monte Carlo sampling, knowledge-based potential models, and these methods with machine learning implementation [85,86,87]. The latter methods with low cost and high throughput are more suitable for virtual screening and drug design purpose, and several CAS applications have been developed using these methods. Barlow et al. implemented the “flex ddG” method in Rosetta macromolecular modeling suite, combining Monte Carlo sampling, torsion minimization, ensemble averaging, and advanced energy functions to achieve higher prediction accuracy [88]. The “backrub” implementation in the flex ddG method considers the conformational flexibility of proteins through sampling rotamers of backbone and sidechain [89]. BudeAlaScan, another advanced CAS application recently developed by Ibarra et al., allows processing structure ensembles (single X-ray and cryo-EM structures, NMR ensembles, and MD trajectories) and considers structural heterogeneity [90]. The authors compared the performance of CAS tools, showing good agreements with experimental values as well as reproducibility among the CAS tools available. Machine learning applications of CAS tools are also developed [91]. mCSM relies on graph-based signatures where predictive models are trained based on encoded distance patterns between atom pairs [92].

All these tools have been great resources when one tries to find key residues on the protein surface for oligomerization where GPCR is no exception. More computational studies for GPCR oligomerization were focused on predicting and targeting interaction surfaces. The following examples utilized MD simulation that is based on force field derived from the nature of physics, granting detailed insights. Shan et al. investigated the ligand-specific oligomerization of GPCR by simulating different types of ligands bound to the GPCR monomer with all-atom MD simulation [93]. The conformational rearrangement of 5-HT_2A_R with various ligands was analyzed, and they concluded that inverse agonist Ketanserin would yield the hydrophobic mismatch-driven oligomerization. Since all-atom MD simulation is too expensive for direct PPI such as oligomerization, other groups utilized coarse-grained MD (CG-MD) allowing long-timescale and rare-event observation. Baltoumas et al. studied GPCR dimer interactions using coarse-grained MD (CG-MD) simulation followed by CAS for interface classification, allowing the detection of hotspots [94]. They investigated a few theoretical dimers based on biophysical evidence, distinguished stable ones with a certain interface from CG-MD, and rationalized the result using CAS and network analysis. Johnston et al. utilized metadynamics, an enhanced sampling method gradually modifying the underlying potential energy, with CG-MD for predicting a more probable dimer interface for two homodimers, β1 and β2-adrenergic receptors [95]. From a total of 160 microseconds of simulation, the authors concluded that the TM1/H8 interface is more favorable than the TM4/3 interface for both dimers.

### 3.2. Application of Artificial Intelligence to Predict PPIs

Identifying a set of amino acids, including conserved residues such as Trp and Arg, at the receptor–receptor interface that may assist the oligomerization process is crucial. Receptor oligomerization has been considered a fundamental molecular mechanism which controls redundancy among GPCRs in various cell types [96]. To clarify the functional and evolutionary mechanisms of oligomerization, primary and tertiary structures of proteins, aggregation propensities of receptors, and free energies of dissociation of complexes have been analyzed [97]. The conservation propensity of Trp is the highest, and Arg has the second highest conservation propensity at binding sites, proving the consistency to experimental data in general [98]. The triplet puzzle theory determines the tendency of GPCRs to form receptor heterodimers. This theory incorporated a common consensus established through experimental and computational works, which demonstrated the tendency of hotspot residues at the protein–protein interface to be protected from the surrounding solvent [78,98]. Exploiting this hypothesis, the triplet puzzle theory gave rise to a series of triplet homologies that were successfully used to infer the propensity of a receptor pair to form GPCR heterodimers [99,100]. These triplet homologies are generally comprised of residues located at the receptor–receptor interface with one residue homology corresponding to a hotspot amino acid pair at the binding interface, and the other residue homologies correspond to neighboring amino acids responsible for obstructing solvent access to the binding hotspot [99]. Therefore, the extraction of a set of deduced triplet homologies assisting receptor–receptor interactions can define a kind of code to predict which receptors should or should not form heterodimers. Based on mathematically rigorous approaches, several triplet homologies located at the receptor–receptor interface were demonstrated to be responsible for GPCR oligomerization [99]. The contact map space consists of residue–residue contacts at the interface between a receptor and a ligand [99]. Thus, machine learning or deep learning applications are required to better understand the contact map space with residue–residue contacts. 

There exist various machine learning and deep learning approaches to identify contact mapping of PPIs [101]. A two-step approach combining Support Vector Machine (SVM) and Mixed Integer Linear Programming (MILP) is utilized; SVM detects contacts, which have higher confidence scores than optimized threshold, and if there are no contacts identified by SVM, MILP is used for protein contact prediction [102]. The random forest method is applied to the recognition of patterns of secondary structure in contact maps by iteratively improving the previous prediction. This method is based on the assumption that the observed residue–residue contacts are clustered with other contacts [103]. Deep convolution neural networks can be utilized to identify more contacts considering distance thresholds to classify contacts and non-contacts. The application of convolution neural networks can predict overall contact maps using multi-layer approaches. These machine learning and deep learning methods help to discover significant patterns in protein data when residue pairs are in contact.

To efficiently and effectively predict sequence-based and/or structure-based PPI interaction to be specifically applicable to the analysis of GPCR oligomeric complexes, deep learning and machine learning algorithms or methods such as maximum likelihood estimation, support vector machine, structural matching, naive Bayesian prediction, and co-evolution can be utilized. BindML, Binding site prediction by Maximum Likelihood, is a method for predicting protein–protein interface residues of a given protein structure using information extracted from its protein family multiple sequence alignment (MSA). Protein residue positions along the MSA with the strongest scoring mutation pattern are predicted as protein interface residues [104]. PPI-Detect, a support vector machine model for the sequence-based prediction of protein–protein interactions, numerically encodes a procedure for the development of a support vector machine model, predicting whether two proteins will interact [105]. PRISM, protein interactions by structural matching, is a tool for large-scale prediction of PPIs and assembly of protein complex structures by conducting structural comparisons of target proteins to known template protein–protein interfaces and processing flexible refinement using a docking energy function [106]. Meta-PPISP, an online tool for the site prediction of PPI, implements linear regression methods, training the linear regression model on a set of 35 non-homologous proteins with cross-validation [107]. The Coev2Net algorithm evaluates the conservation of residues in and around the interface by seeding the co-evolution, simulating co-evolution, constructing a probabilistic graph, and implementing the PPI prediction [108]. SPRINT, an ultrafast PPI prediction of the entire human interactome Scoring PRotein INTeractions, is a new sequence-based algorithm and tool for predicting PPIs by calculating the contribution of similar subsequences to the likelihood of interaction [109]. All these methods include deep learning/machine learning algorithms, which can be applied to analyze PPIs in general and be used to detect a specific set of amino acids in the receptor–receptor interface assisting the oligomerization process (Table 1).

## 4. Design of Interface Interfering Peptides (IPs) to Prevent GPCR Dimerization

Generally, PPI modulators are classified based on (i) their effects on the signaling pathway—antagonist or agonist [101,102,103], or (ii) their binding position—orthosteric or allosteric [104,110,111,112]. Orthosteric modulators are easier to rationalize and predict, whereas allosteric modulation is considered more selective, as these sites are more diversified and less conserved compared to the former [112]. Current approaches of PPI modulator design include small molecules, antibodies, and peptides. However, small molecules—a go-to approach for the design of orthosteric and allosteric ligands—are not considered ideal PPI modulators. This is due to the following characteristics of the protein–protein interface, namely (i) a large binding interface, which is approximately 1500–3000 ${Å}^{2}$ in area as compared to the smaller sizes of orthosteric and allosteric binding pockets (≈300–1000 ${Å}^{2}$), (ii) the flatness of the protein–protein interface, and (iii) the involvement of hotspot residues that are distributed over a wide region [101]. Therefore, to cover the large and flat protein–protein interface, the design of peptide inhibitors are preferred, as they are less likely to cause immune reactions and are of acceptable size compared to antibodies. Additionally, peptide-based inhibitors have been shown to provide considerable affinity and specificity to targeted PPIs [101].

### 4.1. Interfering Peptide (IP) Identification

**Figure 4:**
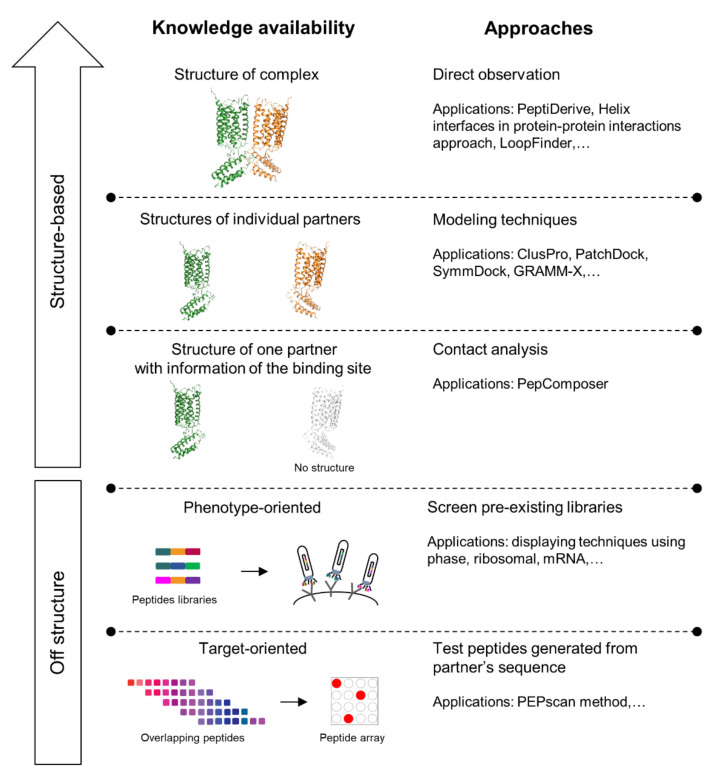
Approaches and applications for interfering peptides (IPs) identification. The arrow on the upper left-hand corner of the figure represents the increasing accuracy of prediction as structure information becomes more accessible.

Peptides have long been used to understand the biological roles of PPIs [113] and have now emerged as potential PPI modulators thanks to their unfavorable pharmacokinetics being improved. Those improvements include improving the proteolytic stability, solubility, cell permeability, and reducing the high level of clearance. The design of interfering peptides (IPs) depends on the availability of the structures of the targeted PPI complexes. Some of the structure-based methods currently used to identify IPs are shown in Figure 4. Advances in in silico tools enabled the comprehensive analysis of the three-dimensional structures of target protein–protein complexes, hence permitting the better understanding of the binding landscape of the protein–protein interface. Additionally, the accessibility to the crystal structure of a protein–protein complex could also assist in the identification of “hotspot residues”—amino acids at the protein–protein interface that predominantly contribute to the interactions between the partnering proteins [114]. Some hotspots at the protein–protein interface have been linked to disease-causing non-synonymous single nucleotide polymorphism (nsSNPs), which is characterized by a point mutation that changes the sequence of encoded protein [104]. 

There are some sequence motifs in GPCRs that could be utilized for the design of IPs, namely Arg-rich motifs and serine-phosphate-containing motif, Small-xxx-Small motifs, and triplet homologies [115]. However, these sequences usually do not show an interfering effect on its own, and chemical modifications such as disulfide bridges, helical modification, and peptide cyclization may be required. The modifications performed are specific to the type of peptides being altered, and this process is made facile through the introduction of IP identification tools. Some examples of these tools include (i) PeptiDerive—a free webserver identifying and evaluating peptide candidates for cyclization using a disulfide bridge [116], (ii) a dataset of helix interfaces in protein–protein interactions as a guide for helix mimetics development [117], and (iii) LoopFinder—a program facilitating the identification of “hot peptide loops” at PPI interfaces [118]. 

The availability of computational prediction tools has also facilitated the design of IPs for protein–protein complexes whereby information are only available for one of the protomers, or only PPIs between the partnering proteins are known [119]. The availability of computational prediction tools, such as protein–protein docking [120,121,122], has also facilitated the design of IPs for protein–protein complexes by allowing the modeling of these complexes even when structures are only available for the protomers individually [123]. When the structure of only one of the protomers with information of the binding site are known, a pipeline named PepComposer can help identify the appropriate sequences in contact with the binding region by analyzing a preexisting contact graph and contact density; then, it can perform the sequence design to propose the potential IPs [124]. 

IPs can be identified with off-structure approaches using in vitro methods, especially when no information of the structure is available. Screening methods have often been applied, using dramatically growing libraries covering both natural and artificial peptides, with the latter generated by scanning the sequence of one of the protein partners or using randomly mixed codons at the interfaces. Some techniques that have been applied for peptides screening could be divided into phenotype-oriented and target-oriented approaches (Figure 4). The former approach includes screening preexisting peptide libraries with display techniques using phase, ribosomal, or mRNA methods with suitable modifications in experiment design with respect to membrane’s effects on GPCRs oligomerization [125]. A target-oriented approach is demonstrated in a technology named PEPscan, which generates a series of overlapping peptides from scanning the sequence of the partner protein. Then, these peptides’ interfering ability are tested in peptide arrays, for which “SPOT synthesis” is considered one of the most popular techniques [123]. Those techniques are now being developed to access millions of sequences for high-throughput screening with improved efficiency [126].

### 4.2. IP Optimization

It is noteworthy that the optimization method highlighted here is unconventionally rigorous. The subject of modification is peptides with a larger degree of freedom and complexity compared to small molecules. The goal is to optimize peptides’ physiochemical properties and ultimately their modulating effects on protein–protein complexes, whose conformational heterogeneity is more frequent compared to one single protein [123]. The objective of optimization is generally to improve both the pharmacokinetics (proteolytic stability, cell permeability) and pharmacodynamics (affinity, selectivity) of the peptides. First, the identification of a minimum active sequence and critical residues is needed to set the starting point for further modification, ensuring that the potency of the parent peptide is optimally retained. Proteolytic instability, one of the major obstacles in IP design, can be tackled by protecting the extremities of the peptide chain, modifying labile amide bonds in the backbone, and substituting key binding residues with analogues containing non-natural sidechains. Sidechain modification can also be conducted on non-critical residues to enhance the solubility of the designed peptides, facilitate conjugation or cyclization [125].

**Figure 5:**
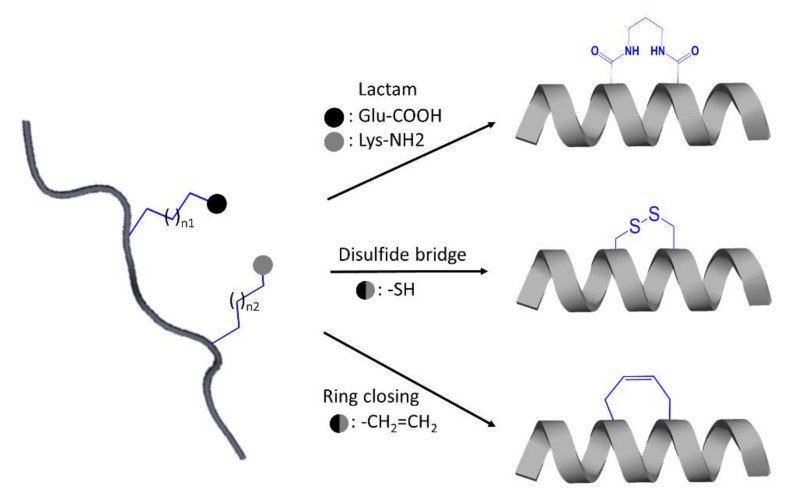
Schematic illustration of some strategies applied for the design of stabilized stapled α-helical peptides.

One of the most promising approaches in peptide optimization is cyclization. When the linear conformation is no longer accessible, the compound becomes more proteolytic-resistant. Furthermore, cyclized conformations, aiming to mimic bioactive conformations with reduced entropic cost when bound to the target protein, acquire increased binding affinity and selectivity [123]. Cyclization typically on alpha-helical peptides can be conducted using the most established method named peptide stapling, which involved the formation of covalent linkage between the sidechains of two amino acids [127]. The amino acids recruited can be natural or non-natural, and the covalently linked amino acid sidechains can be incorporated through several techniques such as the use of lactam scanning to link lysine and glutamic acid sidechains, ring-closing metathesis connecting alkenyl sidechains, and disulfide bridging between two cysteine residues (Figure 5) [123,127]. Even though IP designs mainly target α-helices, designing β-strands, or further, mimicking β-sheet formation from two or more β-strands have been used to interfere with the protein–protein interface [123,125,128]. However, a β-strand is not an ideal motif to mimic due to their high susceptibility to protease degradation. One solution for stabilizing a β-strand motif is the usage of nonpeptidic amino acid analogs [123] as demonstrated in the design of conformationally constraint and stable β-strand using R- and S-bridgehead-substituted β-proline analogues [128]. β-sheet mimetic can be promoted using different strategies, namely the use of turn mimetic, β-strand-enforcing amino acids and macrocyclization [114]. One successful example of a macrocyclic β-sheet peptide is reported in the study of Zheng et al. [129].

Poor cell permeability is another challenge for the design of peptide modulators, including those targeting GPCRs—transmembrane receptors with interactions possibly occurring on both extra- and intracellular interfaces [111,115]. The cell permeability of peptides can be improved by increasing the passive uptake by modulating the hydrophobicity and electrostatic charges of designed peptides (provided that reasonable solubility is maintained) or by increasing its active transport with conjugation to cell-permeable peptides (CPPs). CPPs are generally short, water-soluble peptides that could be linear (majority) or cyclic and are fused to cargoes by covalent or non-covalent bonds [130]. One big challenge for CPP application is the requirement of a high extracellular concentration to ameliorate intracellular uptake level as CPP–cargo fusions are reported to remain within the endosomes [131]. Nonetheless, this is still a useful modification considering its high transduction efficiency and low cytotoxicity [132]. One demonstration for this method is the peptide sequences derived from the D2 dopamine receptor fused with human immunodeficiency virus-type 1 Tat protein (HIV-Tat), which is the most frequently used CPP. These peptides show the antidepressant effect by interfering with the interaction between D1–D2 dopamine receptors [133]. Some bioinformatics tools, namely CPPpred-RF, KELM-CPPpred, and CellPPD, together with CPP libraries such as CPPsite 2.0 are available for in silico prediction and optimization of designed peptides [134].

An interesting example of a peptide modulator designed to disrupt heterodimerization is a stapled peptide comprised of the amino acid sequence of a truncated TM5 of the cannabinoid 1 receptor (CB_1_R) fused to HIV-Tat. This peptide showed a promising disrupting effect on the heteromerization of two GPCRs: CB_1_R and 5-HT_2A_R. The inhibiting effect is dose-dependent, with low micromolar potency (pIC_50_ = 5.47 ± 0.01) and maximal effect reaching over the order of minutes (about 5 min) [135]. This study exemplifies the effect of combining different methods to design an effective and drug-like peptide.

## 5. Concluding Remarks

Recent findings in GPCR oligomerization resulting from protein–protein orthosteric and allosteric interactions further raises GPCRs as attractive therapeutic targets. We have discussed the allosteric modulation mechanism of class C GPCR dimers as well as the dimerization characteristics of class A and C. The trans-activation of well-studied GPCR dimers, the mGluR homodimer and GABA_B_R heterodimer, was explained in detail. The process of understanding GPCR oligomerization with computational aids has been expediated with burgeoning method developments including computational alanine scanning, multi-scale MD simulations, and machine learning applications. Using computational methods of different scales varying from the all-atomistic or coarse-grained level to a knowledge-based model, valuable insights were provided for finding key residues at the G-protein interface, a probable GPCR oligomer when the crystal structure is unavailable, ligands promoting GPCR oligomerization, PPI prediction derived from the protein sequence, etc. With thorough understanding of the interaction between GPCR monomers, attempts were made to develop PPI modulators to hamper the disease pathogenesis by inhibiting GPCR oligomerization. Utilizing a mixture of in silico and in vitro methods, interfering peptides are designed and optimized to increase the pharmacokinetic and pharmacodynamic properties. Since symmetric dimers were mainly observed in crystal structures, the previous studies mainly focused on the homodimers, especially with the same TM domain interfaces. However, the structures of asymmetric dimers and hetero-oligomeric complexes, which existed only as hypothetical computational models with biophysical evidence before, just started to get revealed with enhanced experimental techniques such as Cryo-EM. Benefitting from both experimental and computational advancements, unveiling the oligomeric nature of GPCRs and their functions will take place inexorably.

## Figures and Tables

**Table 1:** List of methods that implement deep learning or machine learning algorithms to predict protein–protein interactions (PPIs).

Method	Description	Website
PPI-Detect	Sequence-based prediction. Based on a support vector machine model trained using pairwise descriptors derived via numerical encoding of the primary sequences of protein pairs embedded as vectors.	https://ppi-detect.zmb.uni-due.de/ (accessed on 22 March 2021)
SPRINT	Sequence-based prediction. Developed based on the assumption that a target protein pair is likely to interact if their subsequence pairs exhibit high degree of similarity with a known interacting protein pair.	https://github.com/lucian-ilie/SPRINT/ (accessed on 22 March 2021)
Coev2Net	Structure-based prediction. Prediction and assessment of individual interactions from a high-throughput experiment. Uses protein threading to generate a homology model of the target, from which extent of co-evolution is calculated.	http://cb.csail.mit.edu/cb/coev2net/ (accessed on 22 March 2021)
PRISM	Structure-based prediction. Uses evolutionary conservation of hotspot PPI residues and considers shape complementarities of protein pairs.	http://cosbi.ku.edu.tr/prism/ (accessed on 22 March 2021)
meta-PPISP	Structure-based prediction of PPI interface residues. Uses scores from three webservers—cons-PPISP, Promate, and PINUP—train a linear regression to predict residues located at protein–protein interface.	https://pipe.rcc.fsu.edu/meta-ppisp.html (accessed on 22 March 2021)
Cons-PPISP	Consensus-based neural network approach for the prediction of residues making up the binding site at the protein interface. Features used to train the neural network include sequence profile and solvent accessibility of neighboring residues.	https://pipe.rcc.fsu.edu/ppisp.html (accessed on 22 March 2021)
Promate	Structure-based prediction of PPI binding sites. Constructed based on quantitative comparison between the PPI interface and other parts of the protein surface in terms of amino acid composition, type of secondary structure, evolutionary conservation, atomic fluctuation, and crystallographic waters.	http://bioportal.weizmann.ac.il/promate/ (accessed on 22 March 2021)

## Data Availability

Not applicable.
